# First Aid Treatment of Jellyfish Stings: A Systematic Review

**DOI:** 10.7759/cureus.84289

**Published:** 2025-05-17

**Authors:** Jen Heng Pek, Swee Han Lim, Gene Y Ong, Therese Djarv, Matthew Douma, Michelle Welsford, Nathan P Charlton

**Affiliations:** 1 Emergency Medicine, Sengkang General Hospital, Singapore, SGP; 2 Emergency Medicine, Singapore General Hospital, Singapore, SGP; 3 Children’s Emergency, KK Women’s and Children’s Hospital, Singapore, SGP; 4 Emergency Medicine, Karolinska Institute, Stockholm, SWE; 5 Critical Care Medicine, University of Alberta, Edmonton, CAN; 6 Emergency Medicine, McMaster University, Hamilton, CAN; 7 Emergency Medicine, Hamilton Health Sciences, Hamilton, CAN; 8 Emergency Medicine, University of Virginia Health System, Charlottesville, USA

**Keywords:** envenomation, first aid, jellyfish sting, pain, systematic review

## Abstract

Jellyfish stings and envenomation can cause local and systemic symptoms with varying severity and duration. The goals of first aid include interventions to relieve symptoms, thus reducing morbidity and mortality. In this systematic review performed on behalf of the International Liaison Committee on Resuscitation (ILCOR) First Aid Task Force, we aimed to answer the question: Among adults and children with a suspected jellyfish sting, what is the effect of first aid intervention as treatment, compared to other treatment or no treatment, on pain, time to pain relief, survival, need for hospitalization, and adverse outcomes or complications? A search for randomized controlled trials (RCTs) and non-randomized studies was performed on Cochrane Central Register of Controlled Trials, Medical Literature Analysis and Retrieval System Online (MEDLINE), Excerpta Medica database (Embase), and Web of Science until 31 March 2025. This systematic review was conducted in accordance with the Cochrane Handbook for Systematic Reviews of Interventions, and reporting adhered to the Preferred Reporting Items for Systematic Reviews and Meta-Analyses (PRISMA) checklist. There were seven RCTs or quasi-RCTs and three non-randomized studies. There was very low confidence in the estimate of effects for the outcomes evaluated due to the risk of bias and heterogeneity. Treatment with topical vinegar was the most frequently evaluated intervention (seven studies), followed by heat therapy (six studies). All studies reported on the critical outcome of any pain relief and intensity of pain, with a wide range of time to pain relief of up to 24 hours. Adverse outcomes after treatment were evaluated in six studies and included pain, erythema, burns, and difficulty with application. Treatments that may relieve symptoms of jellyfish stings include seawater, heat therapy, cold packs, lidocaine, benzocaine, Adolph’s meat tenderizer, vinegar, commercial products (e.g., Stingose or Sting-Aid), and sodium bicarbonate. There were very few studies and a significant risk of bias and heterogeneity, leading to very low-quality evidence on first aid treatment for jellyfish stings. Seawater would be recommended, given its availability at no cost in the coastal region.

## Introduction and background

Jellyfish are found in coastal waters around the world. They have nematocysts, which are specialized stinging cells, and can be triggered by physical or chemical stimuli to fire a barb and release venom. The incidence of jellyfish stings has increased, with more geographical regions being affected due to a rise in jellyfish population; however, this is still an underestimate due to a lack of reporting [1]. Jellyfish stings and envenomation can cause local and systemic symptoms with varying severity and duration. This ranges from immediate pain, local skin reactions such as erythema or urticaria, anaphylactic or delayed hypersensitivity reactions, Irukandji syndrome (characterized by pain as well as autonomic effects such as anxiety, sweating, tachycardia, and hypertension), persistent pain and skin changes such as scarring or hypo/hyperpigmentation, and even death [1]. The goals of first aid include interventions to relieve symptoms, thus reducing morbidity and mortality.

The Cochrane Library released an updated systematic review on the various treatments for jellyfish stings based on six randomized controlled trials (RCTs) and three quasi-RCTs [1]. The authors reported interventions that included hot versus cold applications and topical applications. They had very little confidence in available evidence, and it was unclear if any treatment provided any benefit or harm. In this systematic review performed on behalf of the International Liaison Committee on Resuscitation (ILCOR) First Aid Task Force, we expanded the inclusion criteria of studies to non-randomized trials to provide additional information on the benefit and harm to guide treatment options for jellyfish stings. We aimed to answer the question: Among adults and children with a suspected jellyfish sting, what is the effect of first aid intervention as treatment, compared to other treatment or no treatment, on pain, time to pain relief, survival, need for hospitalization, and adverse outcomes or complications?

## Review

Methods

ILCOR uses a continuous evidence process to evaluate evidence and develop treatment recommendations for relevant first aid topics, culminating in the publication of a Consensus on Science with Treatment Recommendations (CoSTR). This systematic review was conducted in accordance with the Cochrane Handbook for Systematic Reviews of Interventions [2], and reporting adhered to the Preferred Reporting Items for Systematic Reviews and Meta-Analyses (PRISMA) checklist [3].

Selection Criteria

The published articles were selected based on the following: Population refers to adults and children with a suspected jellyfish sting; intervention refers to any treatment (or combination of treatments) to reduce pain or harm that is appropriate for first aid; comparison refers to any other treatment (or combination of treatments) or no treatment; outcomes refer to any pain relief and intensity of pain (critical), time to pain relief (critical), survival (critical), need for hospitalization (important), and adverse outcomes or complications (important). These outcomes were graded through consensus discussion by the ILCOR First Aid Task Force as ‘critical’ or ‘important,’ using the Grading of Recommendations, Assessment, Development, and Evaluation (GRADE) approach [4]. RCT and non-randomized studies (e.g., non-RCT, interrupted time series, controlled before-and-after studies, and cohort studies) were included, while case series, case reports, unpublished studies, theses, conference abstracts, trial protocols, and animal studies were excluded. Studies published in any year and in any language (if an English abstract was present) were eligible.

Search Strategy

Search strings were developed by a librarian for the following databases: Cochrane Central Register of Controlled Trials, Medical Literature Analysis and Retrieval System Online (MEDLINE), Excerpta Medica database (Embase), and Web of Science. The search was performed from the inception date of the databases until 31 March 2025. Refer to Appendix A for search strategies.

Study Selection

Four authors (JHP, GYO, MW, and NPC) independently screened titles, abstracts, and full texts. Reasons for exclusion were documented, and any disagreements were discussed until a consensus was reached.

Data Collection

Three authors (JHP, SHL, and NPC) were involved, with one author extracting the following data from each study: design, population, intervention(s), comparator, and outcome(s).

Risk of Bias and Certainty of Evidence Assessment

The Cochrane risk-of-bias tool for randomized trials (RoB 2) [5] and Risk of Bias In Non-randomized Studies of Interventions (ROBINS-I) tool [6] were used to independently assess risk of bias by two authors (JHP, GYO), and disagreements were discussed until a consensus was reached. The online available Risk-of-Bias VISualization (robvis) tool (https://mcguinlu.shinyapps.io/robvis/) [7] was used to construct risk of bias assessment figures.

The GRADE approach was used to determine the certainty of evidence by assessing the risk of bias, indirectness, imprecision, inconsistency, and publication bias [4, 8]. The final level of evidence was graded as high, moderate, low, or very low.

Data Synthesis

We planned, a priori, to perform random-effect meta-analysis using the generic inverse variance method for continuous data or the Mantel-Haenszel method for dichotomous outcomes and assess heterogeneity by a visual inspection of the forest plot as well as the I² statistic (heterogeneity considered significant if I² > 60%). However, a narrative summary for the outcomes was provided, and no meta-analysis was performed due to considerable heterogeneity in study setting, population, jellyfish species, treatment options, and outcome measures of the included studies.

Results

Literature Search and Study Selection

**Figure 1:**
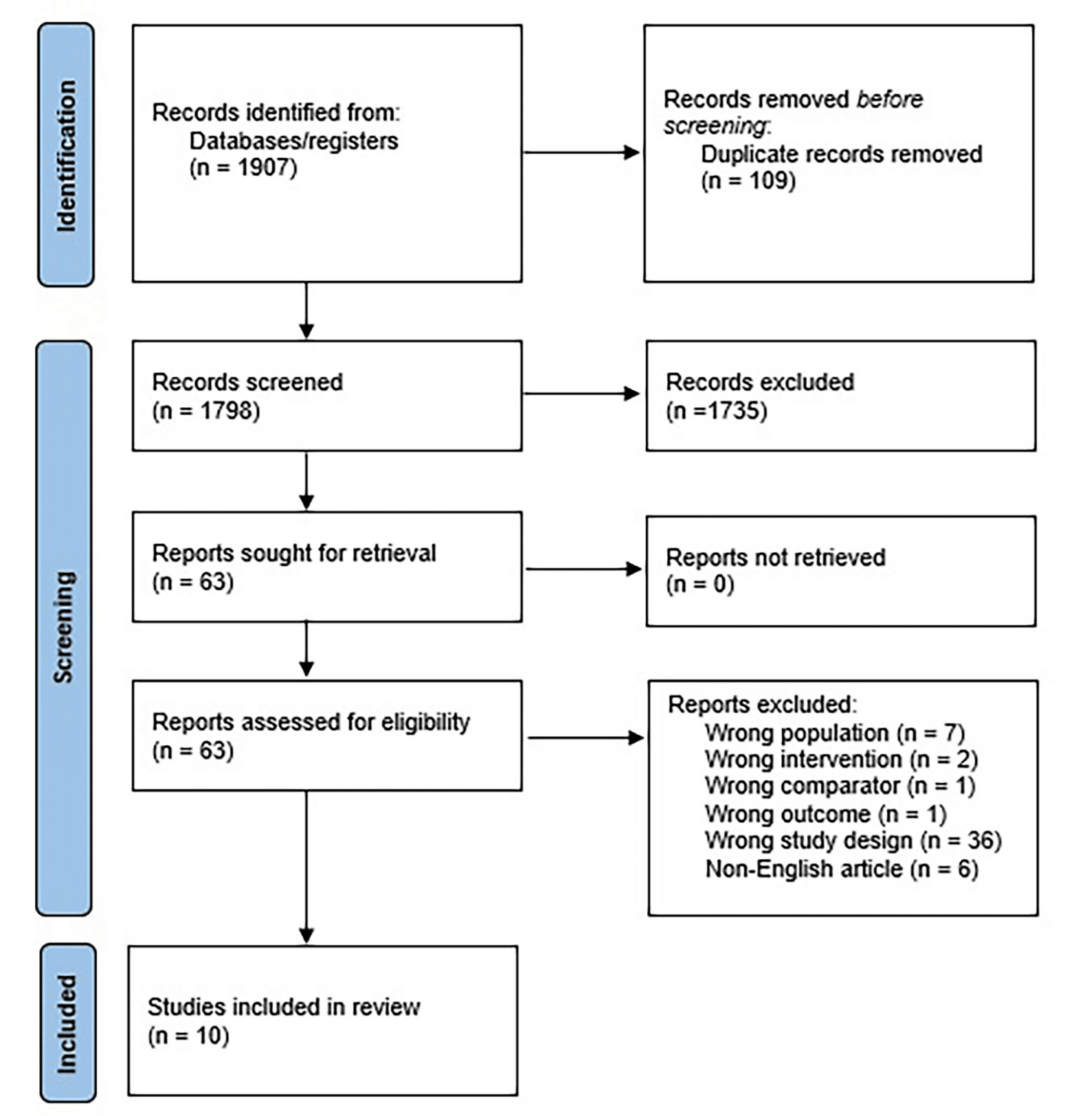
PRISMA diagram outlining the study selection process PRISMA: Preferred Reporting Items for Systematic Reviews and Meta-Analyses

We identified 1,907 references through database search, and a total of 10 studies were included [9-18]. Figure 1 illustrates the PRISMA diagram for study selection.

Risk of Bias and Certainty of Evidence

**Figure 2:**
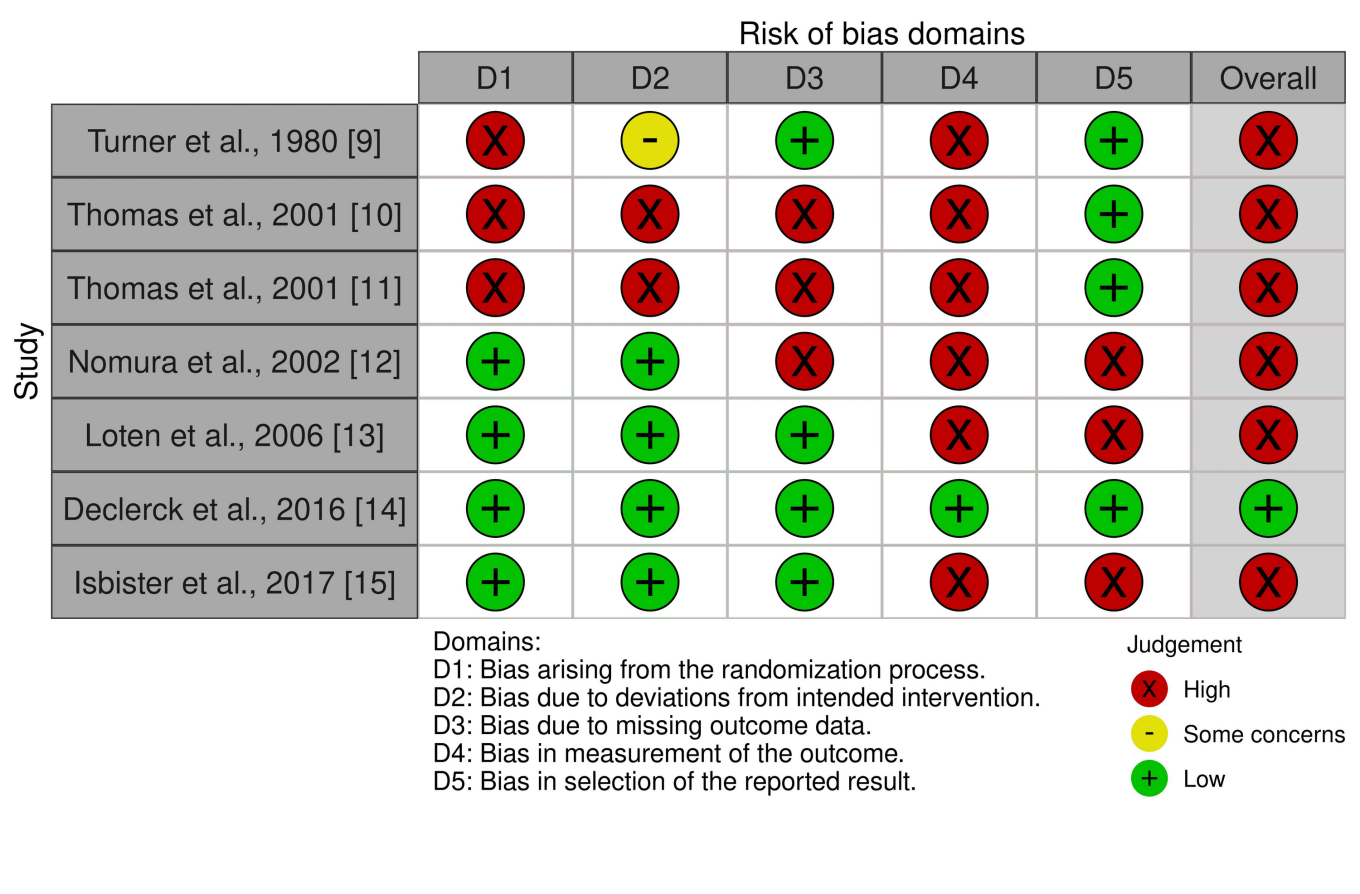
Risk of bias summary for RCTs and quasi-RCTs RCT: randomized controlled trials

**Figure 3:**
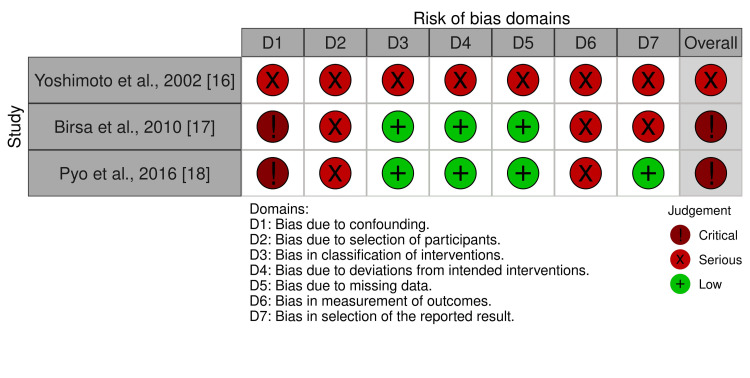
Risk of bias summary for non-randomized trials

We completed risk of bias assessments for the included studies (Figure 2 and Figure 3).

Confidence in the estimate of effect for the outcomes evaluated was very low due to the high overall risk of bias for RCTs and the critical overall risk of bias for non-randomized studies. Biases due to measurement of outcome were most common among RCTs, followed by biases due to the randomization process, missing outcome data, selection of results reported, and then deviations from the intended intervention. Biases due to confounding, selection of participants, and measurement of outcome were common in all three non-randomized studies.

Study Characteristics

Among the 10 studies, there were seven RCTs or quasi-RCTs [9-15] and three non-randomized studies [16-18]. Table 1 shows the study characteristics. These studies originated from three geographical areas, namely, Australia, South Korea, and the United States of America. Five studies [10, 11, 13, 15, 16] enrolled patients with unintentional jellyfish stings. These patients were recruited from lifeguard or first aid facilities at beaches in three studies [10, 11, 13]. Five studies [9, 12, 14, 17, 18] enrolled healthy volunteers who were intentionally inflicted with jellyfish stings. Five studies [10, 11, 13, 15, 16] enrolled children less than 18 years old.

**Table 1:** Study characteristics RCT: randomized controlled trial

Author, year	Country	Study design	Population	Intervention	Control	Outcome
Turner et al., 1980 [9]	Australia	Quasi-RCT	20 healthy volunteers with stings from *Physalia physalis*	Vinegar, Stingose, and methylated spirits	Salt water	Pain and skin reaction at treatment, five minutes and 15 minutes after treatment, by noting sites in order from most to least painful
Thomas et al., 2001 [10]	USA	Quasi-RCT	133 patients (seven years or older) with stings from *Carybdea *species	Vinegar, then chemical hot pack (110°F) or chemical cold pack (42°F)	Vinegar, then the air temperature pack	Pain on a standard 100-mm visual analogue scale at 0, five, 10, and 15 minutes
Thomas et al., 2001 [11]	USA	Quasi-RCT	63 patients (seven years or older) with stings from *Cardybdea *alata	Fresh water, Sting-Aid, and Adolph’s meat tenderizer	Seawater	Pain on a standard 100-mm visual analogue scale at 0, five, 10, and 15 minutes
Nomura et al., 2002 [12]	USA	RCT	25 healthy volunteers with stings from *Carybdea *alata	Adolph’s meat tenderizer, vinegar	Hot water immersion (40- 41 °C)	Pain on a 10-cm visual analogue scale at 0, two, four, six, eight, 10, 15, and 20 minutes, visual appearance of the sting site at 10 to 30 minutes
Yoshimoto et al., 2002 [16]	USA	Observation study	177 patients (15 months or older) with stings from marine cnidaria	Heat therapy with a whole body hot shower, a localized hot pack or a hot wet compress, parenteral analgesic	-	Pain or symptoms of envenoming
Loten et al., 2006 [13]	Australia	RCT	96 patients (eight years or older) with stings from *Physalia *species	Hot water (45 °C) via hose to truncal stings or bucket for limb immersion for 20 minutes	Ice pack (-4 °C)	Pain on a 100-mm visual analogue scale at 0, 10, and 20 minutes, pain reduction as determined by patient description of ‘a lot better’ at 10 and 20 minutes, other symptoms, crossover treatment, itch or rash at 24 hours
Birsa et al., 2010 [17]	USA	Observation study	Two healthy volunteers with stings from *Chrysaora quinquecirrha *and *Chiropsalmus quadrumanus*	Lidocaine, benzocaine, ethanol, acetic acid, and ammonia were applied for one to two minutes	No treatment	Sting sensation and observation of the skin before and after treatment were made by observers
DeClerck et al., 2016 [14]	USA	RCT	97 healthy volunteers (18 to 65 years old) with stings from Chrysaora chinesis	Isopropanol, ammonia, acetic acid, Adolph’s meat tenderizer, lidocaine, sodium bicarbonate, water (40 °C) applied for two minutes, then repeated 15 times for a total of 30 minutes	No treatment	Pain with the initial sting was rated as five, and subsequent measurements were less than or more than five at two-minute intervals for 15 times, 60 minutes, and 24 hours. Erythema was reported as smaller, larger, or the same size, and the andex was created using a computer model.
Pyo et al., 2016 [18]	South Korea	Observation study	Two healthy volunteers with stings from *Nemopilema nomurai* and *Carybdea mora*	Acetic acid, isopropanol, ethanol, lidocaine, distilled water, sea salt solution	No treatment	Sting sensation rated from 0 (no pain) to six (serious burning pain), skin redness rated by observer from 0 (no change) to six (serious)
Isbister et al., 2017 [15]	Australia	RCT	46 patients (eight years or more) with stings from *Chironex fleckeri*	Vinegar followed by hot water immersion (45 ^o^C) for 30 minutes	Vinegar followed by ice packs for 30 minutes	Pain on a visual analogue scale at 0, 10, 20, 30, 60, 90 minutes and on discharge, crossover treatment, use of opioid analgesia, length of stay, development of regional or radiating pain, development of systemic features, urticaria at seven to 10 days after discharge

Treatments evaluated were predominantly via the topical route, with vinegar or acetic acid being the most common in seven studies [9,10,12,14,15,17,18], followed by heat therapy in six studies [10,12-16]. Cold therapy [10,13,15], lidocaine [14,17,18], salt or seawater [9,11,18], and meat tenderizer [11,12,14] (e.g., Adolph’s) were evaluated in three studies. Ethanol [17,18], isopropanol [14,18], commercial products (e.g., Stingose or Sting-Aid) [9,11], ammonia [14,17], and distilled or fresh water [11,18] were evaluated in two studies. Methylated spirit [9], sodium bicarbonate [14], benzocaine [17], and room temperature pack [10], as well as treatment using parenteral analgesics [16], benzodiazepines [16], and epinephrine combined with antihistamine [16], were evaluated in one study.

For the critical outcomes of any pain relief and intensity of pain, they were reported in all 10 studies [9-18]. Six studies [12-17] also reported other outcomes of local or systemic reactions, such as erythema, swelling, anaphylaxis, and Irukandji syndrome.

For the critical outcome of time to pain relief, the studies evaluated a wide range of times after application of treatment, up to 24 hours. Eight studies [9-15,17] reported the time when outcomes were observed.

For the important outcome of adverse outcomes or complications after treatment, it was evaluated in six studies [9,13,14,16-18] and included pain, erythema, burns, and issues with application.

No studies evaluated the critical outcome of death or the important outcome of the need for hospitalization.

Outcomes

Table 2 shows the study findings.

**Table 2:** Study findings 95% CI: 95% confidence interval

Author, Year	Population	Significant findings			
		Pain and pain relief	Local or systemic reactions	Time to pain relief	Adverse outcome of complications
Turner et al., 1980 [9]	20 healthy volunteers with stings from *Physalia physalis*	Compared to salt water, vinegar and Stingose provided significant pain relief (p<0.05) after 15 minutes. There was no significant difference between the efficacy of vinegar and Stingose	-	Pain relief was significant after 15 minutes	Methylated spirits caused a significant increase in pain at the time of application (p<0.01)
Thomas et al., 2001 [10]	133 patients (seven years or older) with stings from *Carybdea species*	Odds ratio for hot pack (110°F) to cease pain was 5.2 (95% CI 1.3-22.8, p=0.02) and odds ratio for cold pack (42°F) to cease pain was 0.5 (95% CI 0.1-2.1, p=0.4). Both hot pack and cold pack had significantly lower average pain scores than the air temperature pack at five minutes. The hot pack had a significantly lower average pain score than the cold pack and the air temperature pack at 10 minutes	-	Hot pack and cold pack ceased pain better than the air temperature pack at five minutes. Hot pack ceased pain better than cold pack and air temperature pack at 10 minutes	-
Thomas et al., 2001 [11]	63 patients (seven years or older) with stings from *Cardybdea alata*	No significant differences in pain score and odds of pain cessation between seawater, fresh water, Sting-Aid, Adolph’s meat tenderizer at 0, five, and 10 minutes (Terminated by authors due to the unlikely that clinical significance would be achieved)	-	Pain scores were assessed at 0, five, and 10 minutes	-
Nomura et al., 2002 [12]	25 healthy volunteers with stings from *Carybdea alata*	Mean difference (1.1, 95% CI 0.6-1.6) of pain scores for hot water immersion (40-41^o^C), and Adolph’s meat tenderizer and vinegar was significant (2.1 versus 3.2, p<0.001) at four minutes Mean difference (1.6, 95% CI 0.9-2.3) of pain scores for hot water immersion, and Adolph’s meat tenderizer and vinegar remained significant at 20 minutes (0.2 versus 1.8, p<0.001), No significant difference between Adolph’s meat tenderizer and vinegar	Visual appearance was rated worse for Adolph’s meat tenderizer and vinegar (16 out of 25 patients) than for hot water immersion (five out of 25 patients)	The mean difference of the pain score, which was significant, was at four minutes and 20 minutes	-
Yoshimoto et al., 2002 [16]	177 patients with stings from marine cnidaria	No difference between heat therapy and analgesics for acute local pain	Heat therapy was superior to parenteral analgesia for the treatment of sting (odds ratio 11.5, p=0.08) - if restricted to a hot shower, the odds ratio was 22.0 (p=0.0485). Heat therapy was superior to analgesic (odds ratio undefined, p=0.40) or benzodiazepine (odds ratio undefined, p=0.10) for the treatment of Irukandji-like syndrome. No difference between heat therapy and a combination of epinephrine with antihistamine for anaphylaxis or anaphylactoid syndrome (p=1.0)	-	Heat therapy was not reported to be deleterious in 60 treatments
Loten et al., 2006 [13]	96 patients with stings from *Physalia *species	Higher proportion of reduce pain among those treated with hot water (45^o^C) compared to ice packs (-4^o^C) - difference of 21% (95% CI 1-39, p=0.039) at 10 minutes, difference of 54% (95% CI 35-69, p=0.002) at 20 minutes Matched difference was 42% (95% CI 19-60, p=0.0005)	Radiating pain occurred less with hot water - difference of 20% (95% CI -39 to -0.003, p=0,039)	Pain relief was at 10 and 20 minutes	No burn from hot water, but transient erythema was common. No adverse effects from the ice pack, but it was difficult to apply continuously
Birsa et al., 2010 [17]	2 healthy volunteers with stings from *Chrysaora quinquecirrha* and *Chiropsalmus quadrumanus*	Immediate pain relief compared to no treatment: lidocaine 10% and 15% produced immediate pain relief, 4% and 5% solutions produced pain relief after approximately one minute, while 1%, 2%, and 3% solutions required 10 to 20 minutes. Increased stinging sensation compared to no treatment: ammonia, ethanol, and acetic acid, no difference compared to no treatment: deionized water, meat tenderizer, and urea benzocaine provided some pain relief but took 10 or more minutes.	Reduced amount of swelling and redness with lidocaine treatment	Pain relief was immediate, at one, 10, and 20 minutes for the different concentrations of lidocaine and benzocaine	Little or no areas of redness were observed after adding lidocaine 4%, 5%, 10%, and 15%. Areas of redness after adding acetic acid or ethanol, or benzocaine
DeClerck et al., 2016 [14]	97 healthy volunteers (18 to 65 years old) with stings from *Chrysaora chinesis*	Adolph’s meat tenderizer less pain on average reported over 30 minutes, 0.46 less than no treatment (p=0.0424)	Sodium bicarbonate reduced erythema index by 12 units (p=0.0013) after 30 minutes. Sodium bicarbonate and Adolph’s meat tenderizer reduced erythema index by 16.5 (p<0.0001) and 8.5 (p=0.0125), respectively, at 24 hours	Pain relief and erythema reduction were at 30 minutes and 24 hours	Ammonia caused first-degree chemical burns
Pyo et al., 2016 [18]	Two healthy volunteers with stings from *Nemopilema nomurai* and *Carybdea mora*	Pain relief for *Nemopilema nomurai* with sea salt solution and lidocaine compared to no treatment. Increased pain for *Nemopilema nomurai* with acetic acid and isopropanol compared to no treatment. Pain relief for *Carybdea mora* with sea salt solution and lidocaine compared to no treatment. Increased pain for *Carybdea mora* with ethanol and isopropanol compared to no treatment	-	-	Erythema for *Nemopilema nomurai* with acetic acid, ethanol, and isopropanol compared to no treatment. Erythema for *Carybdea mora* with ethanol and isopropanol compared to no treatment
Isbister et al., 2017 [15]	46 patients (eight years or more) with stings from *Chironex fleckeri*	11 out of 17 patients treated with hot water (45 °C) immersion and 14 out of 25 patients treated with ice pack application had pain relief at 30 minutes - difference 9% (95% CI -22 to 39%, p=0.75)	Median length of stay was longer with hot water immersion than ice pack application (p=0.07). No patients had recurrent pain or developed hypotension	Pain relief was at 30 minutes	-

For the critical outcome of pain and intensity of pain, hot water immersion was more effective than ice pack application in one study [13] but had no difference from ice pack application in another study [15]. Hot packs and cold packs were more effective than room-temperature packs [10]. Hot water immersion was more effective than Adolph’s meat tenderizer and vinegar [12]. Heat therapy had no difference compared to parenteral analgesics [16]. Sea salt solution, lidocaine, and benzocaine were more effective than no treatment [17, 18]. Ethanol, isopropanol, ammonia, and vinegar resulted in worse pain than no treatment [17, 18]. Deionized water and urea had no difference in pain relief compared to no treatment [17]. Adolph’s meat tenderizer was more effective than no treatment in one study [14] but had no difference from no treatment in another study [17]. Adolph’s meat tenderizer also had no difference compared to vinegar, seawater, fresh water, and Sting-Aid [11, 12]. Vinegar and Stingose were more effective than salt water, but there was no difference between vinegar and Stingose [9].

For the other outcomes of local or systemic reactions, lidocaine reduced swelling [17]. Lidocaine, sodium bicarbonate, and Adolph’s meat tenderizer reduced erythema [14,17], but sodium bicarbonate was more effective than Adolph’s meat tenderizer [14]. Hot water resulted in a better visual appearance than Adolph’s meat tenderizer and vinegar [12]. Hot water reduced radiating pain [13]. Hot water immersion had a longer median length of stay than cold pack application, which was not statistically significant [15]. Heat therapy was superior to parenteral analgesics for the treatment of stings and superior to parenteral analgesics and benzodiazepines for Irukandji-like syndrome [16]. Heat therapy was similar to the combination of epinephrine with antihistamine for anaphylaxis or anaphylactoid syndrome [16].

For the critical outcome of time to pain relief, it was found that while pain relief can occur one minute after treatment [17], any pain relief (if at all) should occur within 30 minutes [14, 15], and the effect can persist at 24 hours [14]. Reduction of erythema can occur 30 minutes after treatment, and the effect can persist for 24 hours [14]. Higher concentrations of lidocaine can lead to faster pain relief [17].

For the important outcome of adverse outcomes or complications after application of treatment, hot water treatment was associated with transient erythema [13] but did not result in burns or deleterious effects [13, 16]. The ice pack was difficult to apply continuously [13]. Methylated spirits resulted in pain [9]. Vinegar, ethanol, isopropanol, and benzocaine in ethanol resulted in erythema [17, 18]. Ammonia resulted in a first-degree burns [14].

Table 3 shows the quality of evidence based on GRADE.

**Table 3:** Quality of evidence based on GRADE GRADE: Grading of Recommendations, Assessment, Development and Evaluation

Outcome	Risk of bias	Indirectness	Imprecision	Inconsistency	Publication bias	Overall quality
Pain and pain intensity	High	Not serious	Serious	Serious	Not serious	Very low
Local of systemic reactions	High	Not serious	Serious	Serious	Not serious	Very low
Time to pain relief	High	Not serious	Serious	Serious	Not serious	Very low
Adverse outcomes or complications	High	Not serious	Serious	Serious	Not serious	Very low

Discussion

The current systematic review provides a comprehensive overview of the evidence on the treatment of jellyfish stings based on ten research articles published as far back as 1980. The inclusion of three non-randomized studies provided further insight with an additional 177 patients and four healthy volunteers [16-18]. These three studies also compared additional treatment options of using heat therapy with parenteral analgesic [16], as well as using ethanol [17, 18], benzocaine [17], and sea salt solution [18] with no treatment. Overall, this review found that while a range of treatment options were available, not all were effective for pain relief and other local or systemic reactions. There may even be adverse outcomes or complications arising from the use of some of these treatment options.

For the purpose of first aid, interventions should relieve symptoms and reduce any further morbidity and mortality as a result of jellyfish stings. The effect of treatment may be brought about by the following mechanisms: removing nematocysts from the affected area, deactivating nematocysts to prevent the release of venom, neutralizing venom injected into the victim, or modulating the sensation or appearance of areas affected by envenomation [1, 19]. However, evaluating the effect of treatment options was more important and pragmatic than determining the exact mechanism underlying treatment options. Similarly, while the effect of treatment options may depend on the species of jellyfish causing the sting, it would not be feasible for the victim or first aid provider to identify the exact species of jellyfish. Therefore, the first aid provider should focus on providing treatment for an intended outcome of relieving symptoms rather than having to consider the mechanism of treatment or identifying the species of jellyfish.

Jellyfish stings are often due to accidents occurring in the coastal waters, which are unplanned and unpredictable. With this in mind, we recommend that the treatment option for first aid should be rinsing the affected area with seawater, as it is more effective than no treatment [18]. Seawater is also readily available at the setting where jellyfish stings occur with no additional cost. When available, heat therapy using irrigation, immersion, application, or a pack and a cold pack may be used after rinsing with seawater, as treatment with fresh water may activate nematocysts [19]. The temperature of heat therapy or a cold pack should be safe to avoid burns and comfortably tolerated by the victim to allow easy application for continuous effect. However, if a first aid facility is available with the ability to stock up on topical treatment options, the use of lidocaine, benzocaine, Adolph’s meat tenderizer, vinegar, commercial products (e.g., Stingose or Sting-Aid), and sodium bicarbonate may be considered. The use of ethanol, isopropanol, and ammonia should be discouraged.

Other than treatment for pain and other local reactions, the first aid provider should also observe for systemic reactions such as anaphylaxis or anaphylactoid syndrome and Irukandji-like syndrome. However, treatment is likely limited on-site, and onward referral to a healthcare facility may be necessary, with activation of emergency medical services for severe or life-threatening conditions. The first aid provider should also be cognizant of possible adverse reactions or complications to the topical treatment, such as pain, erythema, or burns, and manage them accordingly. For victims who improve following first aid treatment, advice on symptoms to monitor for, as well as when to consult medical personnel, should be provided.

Limitations

The main limitation was the inability to perform a meta-analysis, as the studies were too heterogeneous due to the study setting, population, species of jellyfish, treatment options, and outcome measures. Next, while RCTs were identified, a noteworthy drawback was the high overall risk of bias in the conduct of these studies. Previous systematic reviews and meta-analyses, as well as guidelines, were not included in this work. In addition, it was not clear how soon the treatment was initiated after the injury, which may have better effect if initiated earlier. 

## Conclusions

This systematic review identified very few studies that indicated that treatment with seawater, heat therapy, cold packs, lidocaine, benzocaine, Adolph’s meat tenderizer, vinegar, commercial products (e.g., Stingose or Sting-Aid), and sodium bicarbonate may relieve symptoms of jellyfish stings. However, the studies had a risk of bias and heterogeneity. As the quality of evidence was very low, seawater would be the recommended first aid treatment, given its availability at no cost in the coastal region. Future work should focus on time to treatment as well as report long-term outcomes, such as need for hospitalization or survival, and critical outcomes, such as death. 

## Appendices

Appendix A

**Table 4:** Search strategy MEDLINE: Medical Literature Analysis and Retrieval System Online; Embase: Excerpta Medica database

Database	Search strategy performed on 31 March 2025
Cochrane Central Register of Controlled Trials	1. jellyfish*:ti,ab,kw (Word variations have been searched) 2. jelly near/6 fish*:ti,ab,kw (Word variations have been searched) 3. medusa*:ti,ab,kw (Word variations have been searched) 4. MeSH descriptor: [Cubozoa] this term only 5. MeSH descriptor: [Hydrozoa] this term only 6. MeSH descriptor: [Scyphozoa] this term only 7. MeSH descriptor: [Cnidarian Venoms] this term only 8. #1 or #2 or #3 or #4 or #5 or #6 or #7
MEDLINE	1. jellyfish* 2. medusa* 3. cubozoa/ or hydrozoa/ or scyphozoa/ 4. cnidarian venoms/ 5. #1 or #2 or #3 or #4
Embase	1. jellyfish/ 2. poisonous jellyfish/ 3. jellyfish*.mp. 4. (jelly adj6 fish*).mp. 5. medusa*.mp. 6. cubozoa/ 7. hydrozoa/ 8. coelenterate venom/ 9. #1 or #2 or #3 or #4 or #5 or #6 or #7 or #8
Web of Science	((TS=((jellyfish* or cubozoa* or hydrozoa* or medusa* or jelly NEAR/6 fish* or scyphozoa* or cnidarian*))) AND TS=((sting* or poison* or venom*))) AND TS=((therap* or treatment* or relief*))
